# Childhood Poverty Predicts Adult Amygdala and Frontal Activity and Connectivity in Response to Emotional Faces

**DOI:** 10.3389/fnbeh.2015.00154

**Published:** 2015-06-12

**Authors:** Arash Javanbakht, Anthony P. King, Gary W. Evans, James E. Swain, Michael Angstadt, K. Luan Phan, Israel Liberzon

**Affiliations:** ^1^Department of Psychiatry, University of Michigan, Ann Arbor, MI, USA; ^2^Research and Mental Health Services, VA Ann Arbor Health System, Ann Arbor, MI, USA; ^3^Department of Design and Environmental Analysis, Bronfenbrenner Center for Translational Research, Cornell University, Ithaca, NY, USA; ^4^Department of Human Development, Bronfenbrenner Center for Translational Research, Cornell University, Ithaca, NY, USA; ^5^Department of Psychiatry, University of Illinois at Chicago, Chicago, IL, USA; ^6^Mental Health Service Line, Jesse Brown Veterans Affairs Medical Center, Chicago, IL, USA

**Keywords:** poverty, neurocircuitry, amygdala, emotion, emotional faces

## Abstract

Childhood poverty negatively impacts physical and mental health in adulthood. Altered brain development in response to social and environmental factors associated with poverty likely contributes to this effect, engendering maladaptive patterns of social attribution and/or elevated physiological stress. In this fMRI study, we examined the association between childhood poverty and neural processing of social signals (i.e., emotional faces) in adulthood. Fifty-two subjects from a longitudinal prospective study recruited as children, participated in a brain imaging study at 23–25 years of age using the Emotional Faces Assessment Task. Childhood poverty, independent of concurrent adult income, was associated with higher amygdala and medial prefrontal cortical (mPFC) responses to threat vs. happy faces. Also, childhood poverty was associated with decreased functional connectivity between left amygdala and mPFC. This study is unique, because it prospectively links childhood poverty to emotional processing during adulthood, suggesting a candidate neural mechanism for negative social-emotional bias. Adults who grew up poor appear to be more sensitive to social threat cues and less sensitive to positive social cues.

## Introduction

One in four children in the US is now born into poverty (Carsey Institute, 2013). There is strong evidence linking childhood poverty with adverse physical and mental health outcomes in adulthood, irrespective of socioeconomic status (SES) later in life (Wadsworth et al., Forthcoming; Poulton et al., 2002; Adler and Rehkopf, 2008; Shonkoff et al., 2009; Cohen et al., 2010; Birnie et al., 2011). These effects may be explained by the plethora of adverse physical and social conditions confronting disadvantaged children (Evans, 2004). Elevated chronic stress that leads to dysregulation across multiple physiological systems is suggested as a mechanism involved in health inequalities linked with poverty (Evans, 2003). Children from low-income families have higher chronic stress levels, which, in part, are mediated by elevated exposure to cumulative physical and psychosocial stressors (Evans and Schamberg, 2009). Low childhood SES is associated with greater physiological reactivity even to ambiguous social situations as reflected in greater diastolic blood pressure and heart rate reactivity (Chen et al., 2004). Furthermore, poverty is associated with poor mental health (Wadsworth et al., Forthcoming), and childhood poverty increases the risk of major depression, conduct disorder, and PTSD in adolescence (Wadsworth et al., Forthcoming; Kessler et al., 2014).

In addition to exposure to plethora of external stressors, growing up in poverty might also affect individual’s ability to deal effectively with these stressors, by altering their expectations, perceptions, and emotional reactions. Emerging imaging data support links between childhood poverty and anatomical and functional changes in brain areas involved in the induction and regulation of emotion, like the amygdala. The amygdala is engaged in emotional learning, salience and threat detection (Davis and Whalen, 2001; Phelps and LeDoux, 2005), and activation of physiological responses (Knigge, 1961; Herman et al., 1996; Rodrigues et al., 2009). Aberrant amygdala function has been implicated in several anxiety disorders (Etkin and Wager, 2007; Shin and Liberzon, 2010), and linked to a history of lower SES during development. Changes in amygdala volume (Noble et al., 2012; Luby et al., 2013; Hanson et al., 2015) and higher amygdala responses to angry facial expressions (Muscatell et al., 2012) are reported in children and adolescents with lower SES. We recently reported that amygdala reactivity is negatively correlated with childhood family income during emotion regulation task (Kim et al., 2013), in the same cohort of subjects reported here. Gianaros et al. (2008) reported associations between amygdala responses to emotional faces in healthy young adults and their subjective perceptions of parental SES. They interpreted the association between perceived parental low SES and amygdala functional responses as suggestive of negatively biased amygdala response to signs of threat. In support of this interpretation, children from lower SES families were more likely to appraise ambiguous social situations as negative or hostile in intent (Chen et al., 2004). This bias may indicate vigilance and screening for threat as a protective measure.

The amygdala response to emotional stimuli is regulated by prefrontal cortical regions (Milad and Quirk, 2012) with ventrolateral (VLPFC), dorsolateral (DLPFC), medial (mPFC), and rostral anterior cingulate cortex (ACC), all engaged in complex regulation of affect and appraisal of emotional cues (Mayberg, 1997; Etkin et al., 2006; Kalisch, 2009; Ochsner et al., 2012; Prater et al., 2013). Not surprisingly, aberrant function of prefrontal regulatory areas, in addition to the amygdala, has also been linked to abnormal emotional responses and psychopathological states like anxiety disorders (Etkin and Wager, 2007; Shin and Liberzon, 2010). Emerging evidence also has linked lower childhood SES with reduced ACC volume (Cohen et al., 2006), and with reduced activations in ACC, hippocampus, posterior cingulate cortex, insula, and caudate, in adulthood (Silverman et al., 2009). Similarly, lower childhood SES has been linked to lower gray matter volume in adult perigenual ACC (Gianaros et al., 2007), and to lower dorsomedial PFC activation in response to angry faces (Muscatell et al., 2012). The same cohort used in this study, showed reduced VLPFC and DLPFC activity in an emotion regulation task correlated with lower childhood SES (Kim et al., 2013). One mechanistic question that remains unanswered still – is lower SES associated primarily with intrinsic amygdala-based deficits or biases in emotional reactivity/responses, or the reported findings are the results of diminished regulatory functions in cortical regions, while amygdala responses are essentially normal.

In the current work, we sought to examine the prospective relationship between childhood poverty and adult brain reactivity to emotional faces, utilizing task devoid of explicit (or implicit) emotional regulation components. Heightened amygdala reactivity to negative emotional stimuli might underlie negatively toned emotional bias in individual with a history of childhood poverty (toward negative and away from positive social cues). We utilized a unique long-term, longitudinal cohort of poverty, following up participants in early adulthood (23–25 years old), hypothesizing that childhood poverty would be associated with elevated adult amygdala responses to negative emotional faces relative to positive emotional faces. We also expected to observe reduced connectivity between mPFC (emotion regulatory area) and the amygdala in relation with poverty.

## Materials and Methods

### Subjects

Demographic data are shown in Table 1. Fifty two healthy, un-medicated Caucasian subjects, without current or past Axis I psychiatric diagnosis confirmed by clinician-conducted Structural Clinical Interview for DSM-IV, participated in this study. Participants were drawn from a 20-year longitudinal study of rural poverty and child development. Twenty-five of the participants (men and women, mean age of 24.4 ± 1.2 years) spent their childhood in low-income households (income-to-need ratio <1.5 in New York State) and 27 participants (average age 23.1 ± 1.2 years) grew up in middle-income households (income-to-need ratio >1.5). Income-to-need ratio is a per capita index, adjusted annually for costs of living. A ratio equal to or less than 1.0 is defined by the US Census Bureau as “poverty.” The lowest level of education was high school graduate and there was no difference in the level of education between the two groups [*c*^2^(6, *N* = 52) = 8.58, *P* = 0.20]. All subjects were right handed, and none had a major medical illness or contraindication for MRI (e.g., metallic/ferrous materials in their body). This study was approved by the University of Michigan and Cornell University Institutional Review Boards and all participants provided informed consent.

**Table 1:** **Demographic data of the two groups of subjects**.

Group	Number of subjects	M/F	Age
Low SES	25	14/11	24.4 ± 1.2
Mid SES	27	14/13	23.1 ± 1.2

*Low SES: low family of origin income; Mid SES: mid family of origin income*.

### Experimental task

We used a variant of an emotional faces matching task (Hariri et al., 2002). This task has previously been shown to reliably and robustly engage the amygdala and has been widely used to assess amygdala’s reactivity to social cues. Participants viewed a trio of faces on the screen and were instructed to choose one of the two faces on the bottom that expressed the same emotion as the target face on top (Figure S1 in Supplementary Material). The face photographs were selected from a validated stimulus set (Gur et al., 2002). The identities of the three faces were different and overall an equal number of male and female faces were presented. The target and congruent probe faces displayed one of four expressions (angry, fearful, happy, neutral); and the other (incongruent) probe faces always displayed a neutral face (during emotional target blocks) or a pseudo random emotional face (during neutral target blocks). Equal numbers of angry, fearful, and happy faces were randomly presented across trials. Blocks of face matching tasks were interspersed with blocks of a baseline task of matching geometric shapes (circles, rectangles, and triangles) with similar instructions as above, to maintain attention and provide a non-emotion processing contrast. Three blocks of each emotional face were presented, interspersed with blocks of shapes; each block was presented for 20 s.

### Acquisition of MRI data

All scanning was performed using a Philips 3 T MRI scanner (Phillips Medical Systems, Andover, MA, USA) in the functional MRI laboratory at the VA Ann Arbor. A total of 240 T2*-weighted echo planar gradient-recall echo volumes (echo time = 30 ms, repetition time = 2000 ms, 64 × 64 matrix, flip angle = 90°, field of view = 22 cm, 42 contiguous 3 mm axial slices per volume) were acquired. Five additional volumes were discarded at the beginning of each run to allow for equilibration of the MRI signal. A high-resolution T1-weighted structural image was also obtained to provide for more precise anatomical localization.

### MRI data analysis

Data were analyzed using the statistical parametric mapping software package, SPM8 (Welcome Department of Cognitive Neurology, London, UK). Functional volumes were slice time corrected to account for temporal differences in slice acquisition time, realigned to the 10th volume to correct for head motion, segmented into gray matter, white matter, and CSF using the voxel-based morphometry toolbox (VBM8) and spatially normalized to a standard template based upon the Montreal Neurological Institute (MNI) reference brain using DARTEL high-dimensional warping, and spatially smoothed using a 6-mm FWHM Gaussian kernel. Single-subject analysis was performed using standard GLM analysis in SPM8. Models consisted of regressors for task conditions (angry, fearful, happy, neutral blocks) as well as nuisance regressors consisting of the motion correction parameters from the realignment preprocessing step. Contrasts of responses of each facial expression to shapes, and *a priori* defined linear contrasts of interest – threat faces (Angry + Fearful) vs. positive (Happy) faces were generated for each subject, and then entered into a second-level general linear model treating subject as a random effect (random effects analysis). Given evidence suggestive of different processing of angry and fearful threat cues (Whalen et al., 2001, Fusar-Poli et al., 2009) to explore the potential specific signature effects of each of the threat faces (fearful and angry) in comparison to positive faces, we then examined the contrasts fearful > happy, and angry > happy. This approach has previously been effective in isolating threat (angry, fearful) vs. non-threat (happy) social signals while controlling for common face characteristics of the stimuli (Phan et al., 2008, 2013). Regression analysis was performed on contrasts of threat vs. happy faces, using childhood income-to-needs ratios as the predictor of interest (controlled for age, gender, and current income-to-needs). Analyses controlled for concurrent adult income-to-needs to rule out the possibility that childhood poverty findings simply reflect current SES. *A priori* defined regions of interests (ROI) – amygdala and mPFC were examined for differences in emotional activations based on the existing literature (see above). For ROI analyses, small volume correction (SVC) with FWE-corrected *P* value <0.05 was used. Bilateral amygdala and mPFC SVC masks were created using anatomical AAL atlas. To explore direction of the changes contributing to the observed effect in the amygdala in contrast Fearful > Happy (see Results), betas for Fearful > Shapes and Happy > Shapes contrasts were extracted using anatomical amygdala masks.

To evaluate functional connectivity between amygdala (seed region) and other brain regions during processing of emotional faces, we utilized psychophysiological interaction (PPI) analysis, comparing context-dependent connectivity during processing of threat (angry, fearful) faces and the connectivity during processing of positive/non-threatening (happy) faces. Amygdala seeds (10 mm diameter spheres) were created around voxels of maximum activation in left and right amygdala using the all faces > shapes contrast. Deconvolved time series for amygdala seeds from each participant were multiplied by a block vector representing the contrast of interest (Angry > Happy and Fearful > Happy), and individual models contained regressors for the amygdala seed time series, the original conditions, and the interaction terms, and regressors were convolved with the canonical HRF (McLaren et al., 2012.) Resulting contrast maps were entered into second-level random effects regression analysis using income-to-needs ratio. SVC for PPI analysis was performed using the above-mentioned mPFC mask and threshold.

To see the effects of poverty at specific developmental epochs, in a secondary analysis, we also examined the effect of income to need at waves 2 and 3 of the cohort study (mean ages 13.4 and 17.3). Given evidence for chronic stress mediating effects of poverty on brain function during emotion regulation in the same cohort (Kim et al., 2013), we also examined association between the measure of chronic stress and our neuroimaging variables.

## Results

There was no between-group difference in face matching task performance accuracy for each of the faces separately, or for matching of shapes (all *P* values >0.05). As predicted, the Emotional Faces Assessment Task (EFAT) robustly activated amygdala in response to all four facial expressions (Figure S2 in Supplementary Material). Also relative to control condition, a pattern of mPFC deactivation was observed in all four facial expressions (Figure S3 in Supplementary Material). This is not unexpected as mPFC is part of the default mode network and deactivates during tasks that require attention. Of note, these patterns of amygdala activation and mPFC deactivation were observed in both childhood low- and mid-income groups of subjects when examined separately.

### Income-to-need ratio regression analyses

Results of regression analyses of income-to-need ratio are summarized in Table 2.

**Table 2:** **Small volume corrected coordinates, number of voxels, *z* score, and cluster level FWE-corrected *P* values for amygdala and mPFC in correlation with childhood income-to-need**.

Region	Area	*Z* score	FWE *P* value	Cluster size	*x*	*y*	*z*
**Amygdala**
Fearful–happy	L AMYG	3.32	0.014	17	−21	2	−17
**PFC**
Angry–Happy	mPFC	3.77	0.048	68	3	59	4

*Masks are created from MNI anatomical atlas. Correlations are negative. Small volume corrections were done for all the ROIs in all the three contrasts. Only significant findings (*P* < 0.05) are reported in the table*.

#### Amygdala

**Figure 1:**
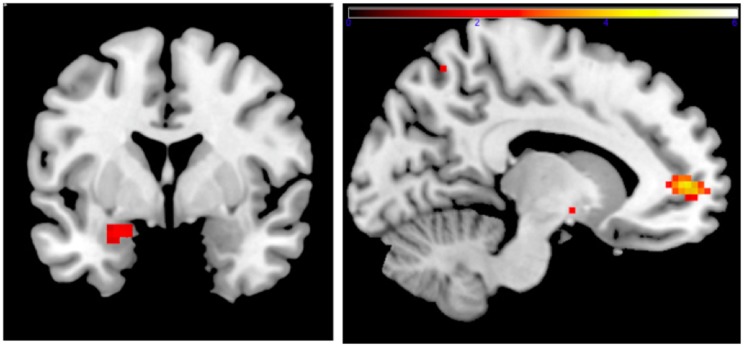
**Left: correlation of adult amgydala response to emotional faces with childhood family income**. Small volume-corrected left amygdala response in contrast Fearful > Happy faces is negatively correlated with childhood income-to-need ratio. Right: correlation of childhood family income and functional connectivity when viewing emotional faces. Small volume-corrected regression analysis of the connectivity between left amygdala and mPFC in contrast Angry > Happy is in correlation with income-to-need. Correlation is positive (coordinates: −12, 53, 4; cluster size 27, peak level FWE-corrected *P* = 0.015; *Z* = 4.14). Same analysis for contrasts Fearful > Happy and Neutral > Happy was not statistically significant.

**Figure 2:**
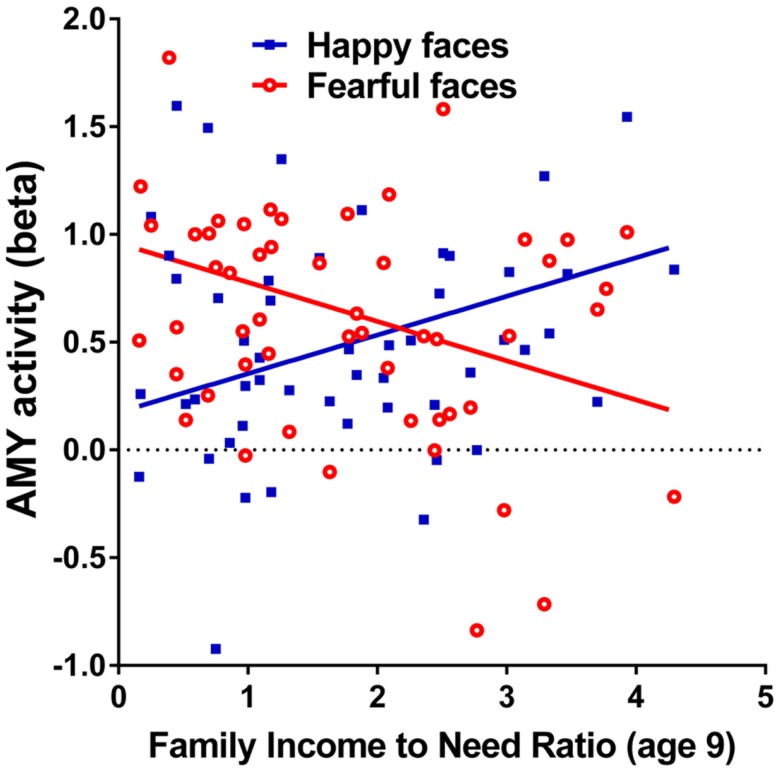
**Correlations of adult amygdala responses to fearful and happy faces with childhood family income**. Relationship childhood income-to-needs ratio (on *x* axis), and amygdala reactivity (on *y* axis) to fearful faces – shapes (open circles) and happy faces – shapes (filled squares) expressed as parameter estimate of extracted from anatomical amygdala ROI (*y* axis). Fearful faces *R^2^* = 0.106, *P* = 0.017; Happy faces *R^2^* = 0.095, *P* = 0.024.

Family income-to-need was negatively correlated with the amygdala activation in contrast threat > happy faces (coordinates: −18, 2, −20; cluster size 11, cluster level FWE-corrected *P* = 0.036; *Z* = 3.52). In further exploration, this effect was found to be due to larger activation in Fearful > Happy contrast (see Table 2; Figure 1, left). Family income during childhood explained a substantial portion of the variance of amygdala reactivity to both Fearful faces vs. Shapes (negative correlation of income-to-needs ratio to amygdala activation parameter estimate, *R*^2^ = 0.10, *P* = 0.02), and to Happy faces vs. Shapes (positive correlation of income-to-need to amygdala activation beta, *R*^2^ = 0.10, *P* = 0.02) as shown in Figure 2.

#### Medial Prefrontal Cortical Region

Peak activation in the mPFC, in contrast threat > happy faces, inversely correlated with childhood income to need ratio (trend level) (coordinates: −3, 29, 40; cluster size 59, cluster level FWE corrected *P* = 0.053; *Z* = 3.39). This effect was due to larger activity in mPFC in contrast Angry > Happy (see Table 2).

#### PPI Analysis

Connectivity between left amygdala (seed region coordinates: −21, −7, −17) and mPFC in the contrast Angry > Happy was positively correlated with childhood income-to-need. Adults with higher income-to-need ratio during childhood exhibited higher amygdala–mPFC connectivity (coordinates: −12, 53, 4; cluster size 27, peak level FWE-corrected *P* = 0.015; *Z* = 4.14) (Figure 1, right).

To examine the possibility of overcorrection of the effects by controlling for current income to need ratio, we also did the analysis without this correction, which revealed similar results (left amygdala Fearful > Happy, coordinates: −24, 2, −20; cluster size 17, peak level FWE-corrected *P* = 0.010; *Z* = 3.41; mPFC Angry > Happy, coordinates: −3, 29, 40; cluster size: 69; peak level FWE-corrected *P* = 0.031; *Z* = 3.90; left AMYG–mPFC PPI Angry–Happy, coordinates: −12, 53, 4; cluster size 28, peak level FWE-corrected *P* = 0.028; *Z* = 3.95).

#### Specific Developmental Epochs

A secondary analysis using the income to need at waves 2 and 3 (mean ages 13.4 and 17.3) did not show the above effects.

#### Chronic Stress

Chronic stress (see Kim et al., 2013) was not significantly associated with the functional neuroimaging findings in the amygdala (*r* = 0.210, *P* = 0.135) or mPFC (*r* = −0.076, *P* = 0.590). Also, current Achenbach anxiety subscale was not correlated with the childhood income-to-need ratio (*r* = 0.026, *P* = 0.857), or the above brain activation findings.

## Discussion

We investigated the link between childhood poverty at age 9 and neural processing of emotional cues during adulthood. Lower SES (as measured by income-to-needs) during childhood was associated with greater amygdala reactivity to fearful faces, and conversely, higher childhood SES was associated with higher amygdala reactivity to happy faces. This is in line with previous findings suggesting negative social bias in people with history of childhood poverty (Chen and Matthews, 2001; Gianaros et al., 2008; Muscatell et al., 2012; Kim et al., 2013). This bias could reflect sensitization to and vigilance for threat cues due to exposure to adverse social environment during childhood in people from lower SES families (Chen and Matthews, 2001). This negative cue sensitization runs in parallel with reduced responsivity to positive emotional cues, which could be due to reduced childhood exposure to such social cues. In other words, there is enhanced perception/response to negative cues and decreased perception/response to positive cues in subjects from lower SES families. Interestingly, the effects of poverty were strongest at the earlier developmental epochs.

These results are in line with previous reports of increased amygdala reactivity to negative or potentially negative social cues (Gianaros et al., 2008; Muscatell et al., 2012). Previous reports have identified increased amygdala activity in people who perceived their parents’ SES as low (Gianaros et al., 2008). This important finding, however, relies on subjective, retrospective perception of parental SES status. As such, the alternative possibility is that subjects who perceive their childhood SES as low might be more sensitive to angry expressions. Our prospective data with objectively assessed poverty early in life strongly suggest that exposure to childhood disadvantage is the likely explanation. While Gianaros et al. (2008) found increased sensitivity to angry facial expression, our study showed a correlation between lower income and stronger amygdala response particularly to fear faces. Amygdala sensitivity to fearful expression, in particular, is consistent with previous literature. For example, Whalen et al. (2001) reported higher amygdala activation in response to fearful faces than to angry faces. In a meta-analysis of 105 studies of neural processing of emotional faces, Fusar-Poli et al. (2009) found that the highest amygdala activation was in response to fearful faces. It has been suggested that fearful faces may evoke more processing due to the ambiguity of the source of threat. In other words, an angry face already provides direct threat cues; whereas a fearful face may require additional processing in order to detect the source of threat (Whalen et al., 2001). As mentioned before, there is evidence in the literature suggesting a bias toward interpreting ambiguous social situations as negative in people from lower SES (Chen and Matthews, 2001). On the other hand, it is also possible that amygdala responses to angry faces were not as high as those to fearful faces because of stronger regulation by higher cortical structures. Indeed, we found that larger mPFC activity in response to angry faces was correlated with lower childhood SES. This result fits with our finding of increased connectivity between left amygdala and mPFC only in response to angry faces. Perhaps, the mPFC is downwardly regulating amygdala responses to threatening, angry faces.

We observed an inverse correlation between mPFC activity and lower childhood income (Angry > Happy faces contrast). Given emotion regulatory function of this brain region, these findings might reflect homeostatic recruitment of mPFC, aimed to modulate the above-mentioned increased amygdala response. Indeed, diminished connectivity between left amygdala and mPFC in lower income subjects observed in response to Angry > Happy faces, in the context of increased activity in both regions, is consistent with less-effective emotion regulation in adults from more disadvantaged childhoods. In other words, in adults who grew up with higher income backgrounds, less mPFC regulatory activity may be required to modulate amygdala responses, perhaps related to both lower amygdala activation and stronger connectivity between the regions. On the other hand, it is possible that higher amygdala responses and lower connectivity in childhood poverty subjects require higher level of activity in mPFC, perhaps signifying less-effective emotion regulation. Alternatively, mPFCs have been implicated in other psychological processes processing self-relevance of stimuli (Kelley et al., 2002; Kalisch, 2009; Moore et al., 2014). It is possible that our finding of higher mPFC activity in response to angry faces in correlation with childhood poverty reflects assessment of negative emotional stimuli as more familiar and self-relevant.

Kim and colleagues reported reduced VLPFC/DLPFC and increased amygdala activation during a reappraisal emotion regulation task in the same cohort (Kim et al., 2013). It is important to note that emotion processing is a large and diverse category of multiple, discrete psychological functions like perception of emotionally salient stimuli, emotional reactivity, cognitive appraisal, and various aspects of emotion regulation (Ochsner et al., 2012). Kim et al. described the effects of childhood poverty on the neurocircuitry involved in reappraisal-related volitional cognitive regulation of emotions, while the current work reports on the differences in implicit emotional reactivity. Accordingly, the neurocircuits involved in these functions are not the same. Specifically, DLPFC and VLPFC are regions involved in selective attention-related emotion regulation and reappraisal (Wager et al., 2008; Ochsner et al., 2012), while the vmPFC, for example, is involved in more implicit forms of emotion regulation (e.g., extinction learning; Etkin et al., 2010). Amygdala is involved in generation of emotional response; however, increased amygdala activation might represent different processes in different studies. In emotional regulation experiments, for example, the increased amygdala signal might represent failure of higher cortical regions to regulate emotional signal. On the other hand, emotional reactivity experiments allow probing more directly amygdala-based processes. Thus, multiple complimentary approaches are required to comprehensively describe complex functions of “emotional processing and regulation.

There are some limitations to this study. Above all, like most other studies of risk factors, this study reports associations that while critically important, should not be interpreted as evidence of causation. The objective assessment and the longitudinal design render important confidence in our findings; however, they do not eliminate the possibility of third explanatory factor associated with both independent and dependent variables. As for the more specific limitations, first, we infer functional effects (emotional response) from amygdala/PFC activity. Indeed, there is a strong body of evidence linking the two (Phan et al., 2004), and we intentionally refrained from asking our subjects to report on their experience – to avoid appraisal processes that could further regulate and alter emotional response. Nevertheless, caution is warranted with this inference. Second, inherent to all fMRI experiments, scanner time is limited, thus using multiple conditions necessarily diminishes potential power. As a result, the condition that most consistently activates given neurocircuitry will be better characterized (Sauder et al., 2013). This could be one of the reasons for detecting stronger responses to fearful faces. Third, mPFC is part of the default mode network, and because the effect of task in this brain region is deactivation, interpreting the differences in the level of activity between contrasts may be difficult. However, because the deactivation main effect was observed in both groups (low and mid SES), the differences observed in threat vs. happy faces seem to be due to less deactivation in the low-SES group. Fourth, generalizability of our findings from viewing emotional expression to experiencing similar emotions and real-life functioning cannot be necessarily assumed given the stimuli used. Although there are similarities in the neurocircuitry involved in perception of emotions in others and viewing emotion-provoking images (e.g., emotional IAPS images), there are also differences. Previous studies have found higher amygdala (Hariri et al., 2002), superior temporal gyrus, insula, and anterior cingulate activation in response to negative emotional faces than negative IAPS (International Affective Picture System) images (Britton et al., 2006). There is also evidence that patterns of brain responses to faces alone may be different than responses to faces in context. The addition of context to faces can provide further information about the possible sources of the emotion expressed in the face and reduce ambiguity. Lee and Siegle (2014) reported larger and more sustained activation in left lateral PFC and mPFC in response to faces presented in context and higher activation in posterior cingulate cortex and left anterior insula in response to faces presented devoid of context. Fifth, in the present study, subjects were only presented with facial expressions. Other communicative elements of real-life experience of others’ emotions (auditory, verbal/cognitive, body language, familiarity) (Kim et al., 2004; Mobbs et al., 2006; Lee and Siegle, 2014) were not explored. Sixth, our study leaves open the question of how underlying neurobiological processes might lead from early-life poverty to the observed neurological functional findings herein. Understanding and experimentally examining these mechanisms will allow to make the critical transition from understanding association to understanding causation. Finally, we did not find significant effects of income to need ratios at waves 2 and 3. Given *N* = 53, the study was powered to detect a correlation of effect size 0.36. Our observed correlation with *Z* = 3.30 produces an estimate of Cohen’s *d* = 0.46; thus, we had sufficient power to detect correlation at the similar level or even lower. Thus, it is possible that we were not powered to detect weaker correlations between brain activity and income at other waves that existed in this sample.

To our knowledge, this is the first prospective study of an objective measure of childhood poverty and neurocircuitry of emotional face processing among adults. Our findings suggest a pattern of sensitization to negative social cues parallel with desensitization to positive cues early in life among poor children, supporting the presence of negative emotional bias in adults raised in lower income (Chen and Matthews, 2001, Chen et al., 2004). Results suggest potentially longstanding negative effects of childhood poverty on adult social-emotional perception and signify the importance of prioritizing attempts toward elimination or reduction of this social problem as early in life as possible to prevent these long-lasting effects. Recall that our prospective findings are independent of concurrent adult income level. While future research can focus on exploration of this bias during childhood (through attentional bias, or eye tracking tasks), prevention of long-term negative outcomes might be even more important. In this context, our findings provide grounds for understanding and advocating for early detection of the possible altered social perception in some children with background of poverty, especially in ambiguous or less safe social situations. Such perceptive differences may lead to negative cognitive appraisal of social situations and less adaptive behavioral response such as avoidance or aggression (Wadsworth et al., Forthcoming). Such maladaptive behaviors likely trigger further negative feedback from the environment and reinforce the biased social perception in these children. Finally, the current study underlines the importance of childhood family income in predicting neural outcomes in young adults during emotion responses. Future studies of remediation strategies addressing these biases, such as enhancement of cognitive skills (Brown et al., 2013) or treatments focused on attention bias modification (Beard et al., 2012; Shechner et al., 2012), across developmental stages may be informed by similar brain-based approaches to reduce long-lasting negative effects of poverty on social, academic, and occupational performance.

## Conflict of Interest Statement

The authors declare that the research was conducted in the absence of any commercial or financial relationships that could be construed as a potential conflict of interest.

## Supplementary Material

The Supplementary Material for this article can be found online at http://journal.frontiersin.org/article/10.3389/fnbeh.2015.00154/abstract
