# Supplementary material for: Childhood Poverty Predicts Adult Amygdala and Frontal Activity and Connectivity in Response to Emotional Faces
Source: Front Behav Neurosci. 2015 Jun 12;9:154. doi: 10.3389/fnbeh.2015.00154 (PMC4464202; doi:10.3389/fnbeh.2015.00154)
Supplement: Supplementary file 1 [file table_1.pdf]

Table S1: correlation matrix of income-to-need ratios in the low-income and the middle income groups and the complete sample

	Low income (N=25)			Mid income (N=27)			Total sample (N=53)		
	**Wave 1**	**Wave 2**	**Wave 3**	**Wave 1**	**Wave 2**	**Wave 3**	**Wave 1**	**Wave 2**	**Wave 3**
**Wave 1**	1	.613**	.500*	1	.328	.400*	1	.683**	.746**
**Wave 2**		1	.445*		1	.504**		1	.710**
**Wave 3**			1			1			1
