# Supplementary material for: Childhood Poverty Predicts Adult Amygdala and Frontal Activity and Connectivity in Response to Emotional Faces
Source: Front Behav Neurosci. 2015 Jun 12;9:154. doi: 10.3389/fnbeh.2015.00154 (PMC4464202; doi:10.3389/fnbeh.2015.00154)
Supplement: Supplementary file 2 [file image_1.pdf]

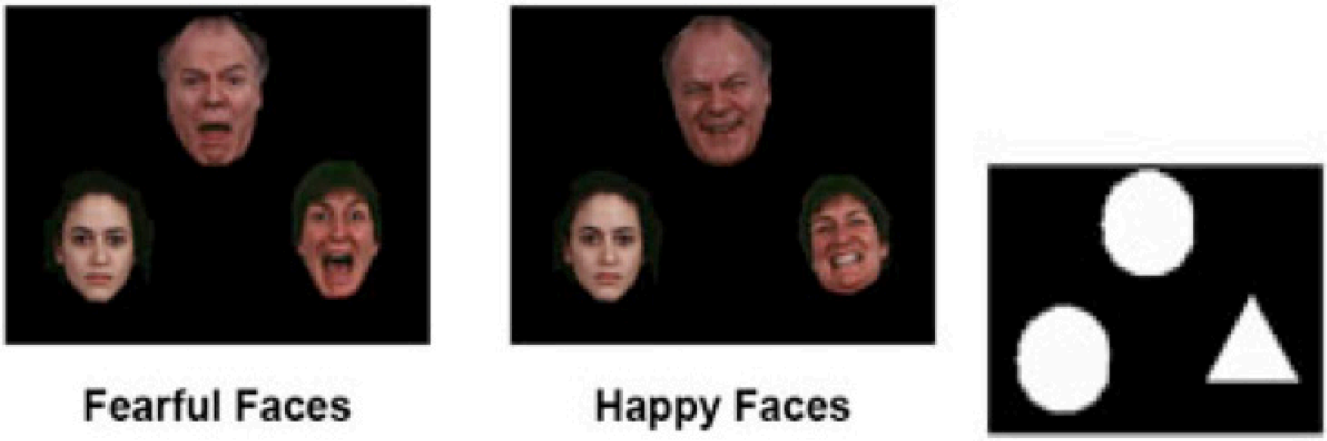

Figure 1 | Emotional Face Assessment Task: Participants viewed a trio of faces on the screen and were instructed to choose one of the two faces on the bottom that expresses the same emotion as the target face on top. the blocks of face matching tasks were interspersed with blocks of a baseline task of matching geometric shapes (circles, rectangles, and triangles) with similar instructions.
