# Supplementary material for: Childhood Poverty Predicts Adult Amygdala and Frontal Activity and Connectivity in Response to Emotional Faces
Source: Front Behav Neurosci. 2015 Jun 12;9:154. doi: 10.3389/fnbeh.2015.00154 (PMC4464202; doi:10.3389/fnbeh.2015.00154)
Supplement: Supplementary file 3 [file image_2.pdf]

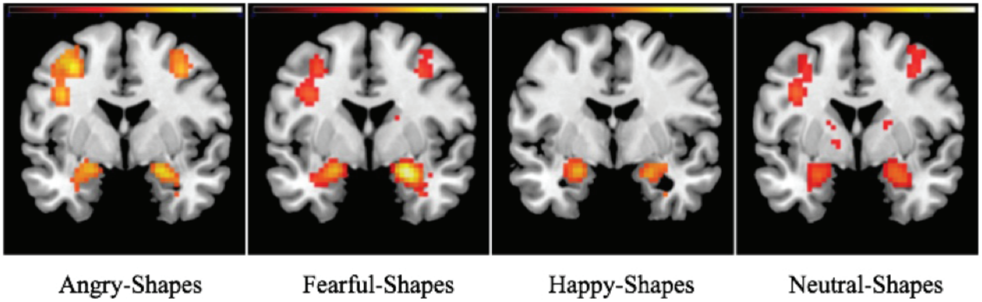


**Figure 2 | Activation of amygdala when viewing images of emotional faces.** Whole brain activation in all subjects in contrasts of faces>shapes at FWE corrected $P<0.05$ shows robust amygdala response to faces with emotional expressions compared to shapes.
