# Supplementary material for: Childhood Poverty Predicts Adult Amygdala and Frontal Activity and Connectivity in Response to Emotional Faces
Source: Front Behav Neurosci. 2015 Jun 12;9:154. doi: 10.3389/fnbeh.2015.00154 (PMC4464202; doi:10.3389/fnbeh.2015.00154)
Supplement: Supplementary file 4 [file image_3.pdf]

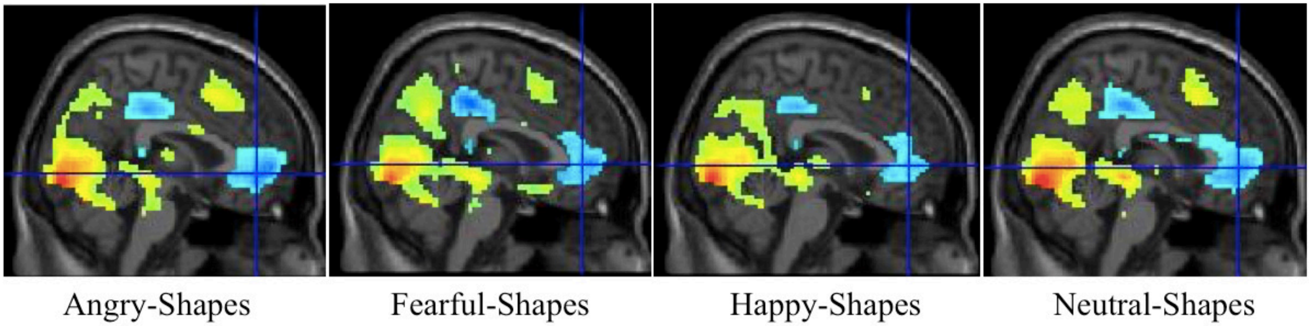

Figure 3 | Deactivation of mPFC when viewing images of emotional faces. Whole brain activation in all subjects in contrasts of faces>shapes at FWE corrected $P<0.05$ shows deactivation in these areas in response to emotional faces compared to shapes.
